# Biofluid lipidome: a source for potential diagnostic biomarkers

**DOI:** 10.1186/s40169-017-0152-7

**Published:** 2017-06-20

**Authors:** Arkasubhra Ghosh, Krishnatej Nishtala

**Affiliations:** GROW Research Laboratory, Narayana Nethralaya Foundation, Narayana Health City, # 258/A, Bommasandra, Hosur Road, Bangalore, 560 099 India

**Keywords:** Lipidomics, Mass spectrometry, Biofluid, Blood, Serum, Plasma, Urine, Tear fluid, Aqueous humor, Cerebrospinal fluid

## Abstract

Lipidomics is the identification and quantitation of changes in the lipidome of a cell, tissue, organ or biofluid in health and disease using high resolution mass spectrometry. Lipidome of a particular organism has relevance to the disease manifestation as it reflects the metabolic changes which can be a consequence of the disease. Hence these changes in the molecules can be considered as potential markers for screening and early detection of the disease. Biological fluids as blood/serum/plasma, urine, saliva, tear and cerebrospinal fluid, due to their accessibility, offer ease of collection with minimal or no discomfort to the patient and provide a ready footprint of the metabolic changes occurring during disease. This review provides a brief introduction to lipidomics and its role in understanding the metabolic changes in health and disease followed by discussion on the chemical diversity of the lipid species and their biological role, mammalian lipids and their metabolism and role of lipids in pathogens and the immune response before dwelling further into importance of studying lipidome in various biological fluids. The challenges in performing a lipidomic analysis at the experimental and data analysis stages are discussed.

## Introduction

Lipidomics involves studying and identifying the structure and functional role of lipids in various cells, tissues and various biofluids produced by all living organisms. Lipids are structural components of the cell membranes and are involved in several metabolic pathways [1, 2]. Lipid metabolism plays important roles in several metabolic disorders including diabetes, metabolic syndromes, other systemic diseases like cancers (e.g. lung cancer, prostate cancer, breast cancer, oral cancers etc.) [3, 4], neurodegenerative diseases (e.g. Alzheimer’s disease [5] and infectious diseases [6] besides their role in regulating autophagy [7], apoptosis and aging [8–10]).

Traditional methods of lipid characterization such as thin layer chromatography (TLC), gas chromatography (GC), nuclear magnetic resonance spectroscopy (NMR) were limited by lack of sensitivity and accuracy [1]. The advent of high resolution, high sensitivity and high mass accuracy mass spectrometers have made possible the identification of multiple lipid species in a given sample. Besides, the introduction of soft ionization techniques such as electrospray ionization (ESI) and atmospheric pressure chemical ionization (APCI) improved the ionization and fragmentation of lipid molecules thereby resolving complex mixtures of lipids [11] and identification of unique constituents.

Systematic reviews on the lipidomics earlier have focused extensively on different classes of lipids, the biological role and functions of lipids, extraction procedures and the advances in mass spectrometry in identifying and quantifying lipids [1, 11–15] from various tissues and in vitro cultured cancerous cells. Biological fluids as blood/serum/plasma, saliva, urine, cerebrospinal fluid, tear fluid, aqueous humor, amniotic fluid etc. also comprise of lipid moieties and can provide information on the disease related changes in the lipid profile. These biological fluids provide an added advantage of mostly non-invasive methods of collection (with exceptions of CSF, blood and amniotic fluid) with minimal or no discomfort to the patient. Hence the focus of the current review is to enable and inform the clinician researcher on the importance of biofluid lipidomics in studying and understanding disease related metabolic changes and how mass spectrometry based lipidomics can facilitate in identifying essential lipid molecules involved in disease processes. Such lipid moieties are of relevance as disease biomarkers or as drug targets. We therefore discuss the importance of the reported lipidomes in individual disease pathologies and underlying molecular functions from biological fluids.

## Review

### Structural diversity of the lipidome, their biological and functional role

Lipids are very diverse in their structure and are classified into eight major classes by International Lipid Classification and Nomenclature Committee into glycerolipids, glycerophospholipids, fatty acyls, sphingolipids, polyketides, saccharo lipids if formed by carbanion condensation of thioesters and prenol lipids and sterol lipids when formed by cabo-cation condensation of isoprene units with each category further subdivided based on the length of fatty acyl chains or the number of double bonds in the hydrocarbon backbone resulting in hundreds of thousands of lipid species [12, 16, 17]. This structural diversity also brings about wide variations in their physicochemical properties and thereby help define tissue/cellular molecular functions. Lipids have multiple roles such as in energy metabolism and storage, structural/barrier function as membrane lipids, cellular signaling, cell proliferation and survival [12, 18–20], calcium homeostasis, membrane trafficking [21, 22], migration of immune cells [23, 24], aging and apoptosis mechanisms [8–10] and autophagy [7, 25].

The above discussed diverse functions of lipids and more make it only rational and important to study the changes in the lipidome of a cell, organ or tissue to understand a disease phenomenon. A significant number of ailments have been associated with altered lipid metabolism [26–31]. Fatty acid synthase and insulin response in obesity has been reported to be controlled by lipid metabolites [32–34]. Reduced triglyceride (TG) utilization caused by digestive lipase inhibition is followed as a treatment for obesity [35]. Plasma sphingosine-1-phosphate levels were found to be elevated in rat and mouse models of diabetes [36]. MALDI-MS lipidomic analysis of the healthy and tumor lung tissue of high MYC activity mice revealed increasing signaling precursor phospholipids–phosphoinositides in tumor tissues while the surfactant lipids were predominant in healthy tissue. Increase in phosphatidyl cholines 32:0, 32:1 and prostaglandins was observed in control tissue compared to tumor [37] which can be due to the alveolar damage in the tumor lung tissue. Comparison of glycerophospholipids among non-small cell lung carcinoma (NSCLC) tissues and normal tissue samples using MS based phospholipidomic approach revealed elevated levels of phosphoinositides PI 38:3, 40:3, 38:2 in tumor tissue samples and decreased levels of sphingomyelins SM 40:1, 42:1, 36:1 compared to healthy tissue [38]. Phospholipids PS 18:0/20:4, PI 18:0/20:4 and PC 18:0/20:4 were identified to be higher in highly metastatic MDA-MB-231 cells than MCF-7 cells [39] indicating altered lipid metabolism in cancer. Changes in the lipid metabolism in various diseases as oral cancer, Alzheimer’s etc. are discussed further in the manuscript in lipidomics of biological fluids.

### Mammalian lipids and metabolism

The cellular metabolic status can be readily understood by studying lipids. The metabolism of the lipids can alter with changes in daily routine such as exercise or either by developing a disease. These alterations involve multiple metabolic pathways and understanding the interplay between these pathways helps in understanding the cellular metabolism. Biologically active lipids such as eicosanoids, prostaglandins, leukotrienes are derived from fatty acids and are involved in the formation of sphingolipids, sterols and glycerols [12]. Sphingolipids are membrane lipids involved in signaling mechanisms. Defective metabolism of sphingolipids is associated lysosomal disorders [40]. Sphingosine and sphingosine-1-phosphate regulate the immune cells and exert receptor mediated function in cells [23, 24, 41]. Phospholipids are another class of membrane lipids which act as precursors for various secondary messengers. For example, phospatidic acid (phosphorylated diacylglycerol) is an intermediate of lipid metabolism (phospholipid biosynthesis) and regulates enzyme function [42, 43].

Phospholipids, especially phosphoinositides (PIs), are signaling lipids and are involved in various cellular processes. The lipid interactions with other molecules (protein–lipid interactions) provides new insights into dynamics of lipids and their mediated responses within the cells. e.g. Pleckstrin homology domains PX and FYVE domains [44]. These various interactions of lipids and their molecular cross talk is essential in maintaining cellular homeostasis and studying these alterations/perturbations in the lipid metabolism that might happen in a disease can help understand the metabolic state of the individual.

### Lipids in pathogens and the immune response

Lipids play a regulatory role in invasion, persistence, replication and immune response to parasitic bacteria [45, 46]. Using molecular mimicry, virulent bacteria as *Mycoplasma* and *Salmonella* interfere with the host lipid metabolism to effectively invade and replicate in the host [47]. Viruses, on the other hand, use specialized membrane microdomains abundant in cholesterol, phosphoinositides and sphingolipids to facilitate entry into the host [48–52] cells. Docasonoid and eicosanoid lipids, termed as “lipid mediators” [53–56] have an important role in inflammation [57]. Protectin-D1, a docasonoid derivative, was shown as a potent inhibitor of H1N1 influenza A infection in infected mice [6]. Arachidonic acid, a precursor of eicosanoids, has also been reported to have a significant role in inflammation [57]. Taken together, mediator lipids can act as molecular modulators or as biomarkers for infection.

### Lipidomics of biological fluids

Biological fluids as blood/serum/plasma, urine, cerebrospinal fluid are the primary sources of lipids besides tear fluid and aqueous humor which provide readily accessible molecular fingerprint reflecting the state of the disease or therapy. Besides, the advances in technological platforms such as liquid chromatography and high resolution, high accuracy mass spectrometry facilitate large scale and high throughput analysis of the lipid molecules.

#### Blood/serum/plasma lipidomics

The lipidome of blood is a complex mixture comprising of phospholipids (PL) [phosphatidylcholines (PC), phosphatidylethanolamines (PE)], sphingolipids (SL) [sphingomyelin (SM) and ceramides (CM)] besides cholesterol esters, triacylglycerols, etc. [58–60]. Quehenberger et al, reported over 500 distinct lipid molecular species quantitatively identified in human plasma. Glycerophospholipids (GP) and sphingolipids (SL), each over 200, constituted the major classes identified in plasma besides sterols and prenol lipids indicating GP and SL are the most abundant of the lipid classes [61]. Serum lipid profiling of patients with hepatocellular carcinoma by UPLC-MS identified glycerophosphoserine (PS(O-16:0/20:2), PS(O-18:0/22:6), PS(O-18:0/22:4)), glycerophosphocholines (PC(15:0/20:3)) and glycerophosphoinositol (PI(O-16:0/20:1)) which can be potential biomarkers for distinguishing hepatocellular carcinoma (HCC) from liver cirrhosis (LC) and chronic hepatitis B (HBV). These four lipids were reported to show higher sensitivity and specificity in HCC (78.1% sensitivity/63.6% specificity) to LC and (93.8% sensitivity/80.0% specificity) to HBV than conventional alpha-fetoprotein (38% sensitivity/93% specificity) indicating the diagnostic potential of mass spectrometry based lipidomic analysis [62]. In case of rare genetic disorders such as Gaucher disease (GD), caused by accumulation of ceramide lipids (monohexyl ceramide—MHC), 125 species of plasma lipids were identified by nLC-ESI–MS/MS of which 20 plasma lipid species belonging to the classes PL and SL were observed to be significantly higher/lower in patients compared to healthy controls. A urinary lipid analysis performed in the same patients identified about 105 lipid species of which 10 lipid species were found to have altered in GD patients compared to controls. Though majority of the lipid species were found in both plasma and urine, their levels differed and certain lipid species were found either in plasma (d18:1/14:0—SM) or in urine (d18:1/20-0-MHC). Lipid analysis performed after enzyme replacement therapy for treatment of GD showed reduced levels of accumulated MHC species which can serve as diagnostic markers for GD in plasma and urine [63].

Serum PL species SM 16:0/1, LPC 18:1, 20:3, 20:4 and 22:6 were identified to have altered in patients with lung tumor which can be used as diagnostic markers for lung cancer [64]. Two recent comparative lipidomic studies on serum of patients with prostate cancer identified phospholipids—egg Phosphatodylcholine ePC 38:5, 40:3 and 42.4 in 154 subjects (77 normal/77 prostate cancer) [65] and PC 36:9 and fatty acid FA 22:3 [66] in 36 subjects (18 normal/18 prostate cancer) respectively, which may act as diagnostic markers for early screening and detection prostate cancer. The authors predict the combination of three lipid species ePC 38:5, 40:3 and 42:4 can be used at 95% confidence to distinguish normal from cancer status at cutoff values of ePC 38:5 ≥ 0.015 nmole, PC 40:3 ≤ 0.001 and PC 42:4 ≤ 0.0001, then the individual is normal. However, if ePC 38:5 ≤ 0.015 nmole and PC 40:3 and 42:4 ≥ 0.001 and 0.0001 respectively, it is very likely to be prostate cancer [65]. A comprehensive analysis of the different cancer related lipid biomarkers has been extensively reviewed by Perrotti et al. [3].

#### Salivary lipidomics

The ease in availability, sampling and especially the relevance in pursuing as a source of biomarkers in oral cancer [67–70], diabetes [71, 72] and chronic oral inflammatory conditions such as periodontitis [72, 73] makes saliva an important biofluid to study disease associated changes in lipid profile.

Majority of the salivary lipids comprise of cholesterol, cholesteryl esters, mono-, di- and triglycerides and free fatty acids. Phospholipids form only a minor fraction of the total salivary lipids [74]. A correlative study of serum and saliva performed in about 100 healthy individuals showed moderate correlation of total cholesterol, triglycerides, high density, and very low lipoprotein cholesterol between the serum and saliva emphasizing the use of saliva as a non-invasive diagnostic fluid for lipid analysis [75]. Lipid analysis of parotid saliva among two groups of female subjects susceptible to- and resistant to dental caries showed higher total lipid concentration in caries susceptible group though the lipid composition essentially remained the same with majority of neutral lipids followed by glycol- and phospholipids. Specifically, neutral lipids, free fatty acids and triglycerides were observed at higher concentrations caries susceptible group and are probably associated with the development of caries [76]. Along with total lipid content, an increase in total salivary protein was also observed in caries susceptible group of patients.

Salivary lipids also play a significant role in oral cancers especially in screening and early detection of the disease. Salivary sialic acid has been studied as a potential screening and diagnostic marker for oral squamous cell carcinoma (OSCC). Higher levels of salivary free and protein bound sialic acid along with total protein and total sugar was observed in unstimulated saliva of patients with OSCC compared to heathy controls (n = 30 each). Significantly higher levels of free sialic acid was observed in well differentiated OSCC patients than the moderately differentiated. Moreover, the levels of sialic acid were reported to correlate very well with histopathological degree of the disease [69]. A similar study performed among control, oral premalignancy and oral squamous cell carcinoma (OSCC) patients (n = 30 each) also identified significantly higher levels of free sialic acid in OSCC and correlated with the grades of the disease in both OSCC and grades of dysplasia in premalignancy [68] indicating the role of free sialic acid as a screening and early detection marker for oral squamous cell carcinoma. Shetty et al., performed a cross-sectional study to evaluate the levels of salivary malondialdehyde (MDA) involved in lipid oxidation among healthy subjects (HC), potential malignant disorders (PMD) and oral squamous cell carcinoma (OSCC) patients. The healthy controls (n = 65) were further sub-grouped into HC1—without quid chewing/tobacco use and HC2—with quid chewing/tobacco use. Also the PMD group (n = 115) is further divided into an another lipid biomarker that has been identified at significantly higher levels in healthy subjects with oral submucosal fibrosis (OSMF) (n = 65) and oral leukoplakia (n = 50). Elevated levels of MDA was observed consistently in HC2, PMD and OSCC groups compared to HC1 [67]. These results indicate elevate lipid peroxidation in oral carcinomas and suggest the role of MDA as a diagnostic marker for detection of PMD and OSCC.

Malondialdehyde as a measure of lipid peroxidation has also been studied in chronic oral inflammatory conditions such as periodontitis, a major cause of tooth loss. A cross sectional study performed to assess the effect of smoking on periodontitis in the saliva of healthy subjects and periodontitis patients (n = 30 each with n = 15 smokers in each group) showed significantly elevated levels of MDA in saliva of periodontitis patients who are smokers compared to non-smoking controls [77]. Glutathione peroxidase (GSHPx), an anti-oxidant enzyme was also observed at elevated levels in periodontitis patients compared to healthy controls. Increased levels of MDA and GSHPx in smoking periodontitis patients indicate elevated lipid peroxidation in periodontitis and is further enhanced by smoking. Using targeted lipidomic approach, Huang et al., identified elevated levels of prostaglandins E2 (PGE2), D2 (PGD2), F2α (PG F2α) in patients with periodontitis but non-smokers and healthy controls (n = 50 each). Elevated levels of salivary F2-isoprostanes which are free radical peroxidation products were observed in periodontitis patient saliva. These data from saliva collectively indicate significant redox alteration and fatty acid metabolism in periodontitis [73].

#### Urinary lipidomics

Urine as an important source of disease biomarkers has been well established and is extensively used in diagnostic medicine [78]. Rockwell et al., have reported reproducible quantitation of over 600 lipid species over 20 lipid classes comprising of both structural lipids and mediator lipids from 0.5 mL of urine using MS/MS^ALL^ shotgun lipidomic workflow wherein all lipid precursor ions are fragmented without any prior selection thereby increasing the possibility of identifying multiple lipid species [79]. Elevated levels of triglycerides (TG), phosphatidylcholines (PC), phosphatidylethanolamine (PE), phosphatidylserine (PE) and free fatty acids were observed in patients with nephrotic syndrome [80]. A more recent study on urinary lipidome of healthy individuals selected across age, sex and body mass index (BMI) revealed that sex and not age or BMI can be a confounding factor in determining the urinary lipid composition in healthy individuals [81]. Sixty urine samples analyzed from healthy white individuals using targeted lipidomics by multiple reaction monitoring (MRM) showed higher anti-inflammatory omega-3 12-lipooxygenase (LOX) oxylipin in women than in men and higher levels of anti-hypertensive oxylipins than in younger men. The implementation of targeted lipidomics approach by MRM in such a large cohort of patients, will enable to evaluate the sensitivity and specificity of the lipid biomarkers and can help in development of more reliable urinary lipid biomarkers [81, 82].

In women, breast cancer is one of the leading causes of cancer related mortalities [83]. Lipidomic analysis of urine from patients with breast cancer showed significant increase in phospholipids—phosphatidylserine (PS) and reduced phosphoinositol (PI) compared to healthy controls. PS species (18:1/18:1 and 18:2/18:0) levels increased in breast cancer patients and were observed to have reduced post-surgery indicating that PS can be used as early diagnostic markers for breast cancer [84]. Similarly in case of prostate cancer, PS species PS 18:0/18:1, PS 16:0/22:6 were observed at higher levels whereas PS 18:1/18:0, PS 18:0/25:0 were observed as reduced in patients compared to healthy controls [85]. Urinary exosome analysis from prostate cancer patients (n = 15) showed quantitatively significant differences for PS 18:1/18:1 and lactosylceramide (d18:1/16:0) compared to controls (n = 13). These lipid species along with PS 18:0–18:2 were observed to distinguish prostate cancer patients from healthy controls with 93% sensitivity and 100% specificity [86] emphasizing the role of urinary lipidomics in biomarker discovery for relevant diseases.

#### Cerebrospinal fluid lipidomics

Cerebrospinal fluid (CSF) is regarded as a promising source of biomarkers in neurodegenerative diseases as Alzheimer’s, dementia and chronic neurological diseases due to its anatomical proximity to the brain and can reflect disease dependent biochemical changes. However, studies examining the changes in lipids as biomarkers in CSF are limited. LCMS analysis of glycerophospholipids (GP) and sphingolipids (SL) from fractionated CSF (supernatant and nanoparticle fractions) among cognitive healthy subjects with normal β amyloid/total tau (CH-NAT) and pre-clinical Alzheimer’s—like β amyloid/total tau pathology (CH_PAT) showed higher levels of certain classes of GP in the supernatant fraction of CH_PAT whereas levels of SL species were observed to be high in nanoparticle fraction of CH_PAT indicating remodeling of high turnover of GP and SP in CSF of preclinical Alzheimer’s patients. Targeting these lipid pathways might help in the prevention of Alzheimer’s [87]. Altered lipid metabolism characterized by elevated levels of ceramide-1-phosphate content in CSF was reported in amyotrophic lateral sclerosis (ALS) besides Alzheimer’s indicating the similarity among various neurological conditions [88]. Elevated levels of CSF phosphocholines (PC) and lower lysoPC/PC ratio is observed in Alzheimer’s patients [89, 90]. Sphingomyelin levels were also reported to be elevated in prodromal stages of Alzheimer’s compared to control [91]. In patients with dementia, Han et al., reported around 40% reduction in levels of CSF sulfatide compared to controls. Besides, sulfatide/PI (Phosphoinositide) ratio has been suggested as a probable diagnostic marker for Alzheimer’s disease with 90% sensitivity and 100% specificity [92].

#### Tear fluid/aqueous humor lipidomics

Tear fluid is a very complex mixture of numerous proteins, peptides, lipids, metabolites etc. Tear fluid acts as a signboard either to understand the etiology of the ocular disease and/or to track the prognosis/response to treatment. Lipids, besides proteins are one of the major components of the tear film with an inner mucin layer, middle aqueous layer and the outer lipid layer. The lipid layer provides lubrication, stability to the tear film and plays a protective role by preventing cornea from drying and shields against pathogens. Rantamaki et al., analysed tear lipidome using thin layer chromatography, enzymatic and mass spectrometric methods and show polar lipids, majorly phospholipids and that the higher percentage of polar lipids is essential for spreading the lipid layer in presence of non-polar lipids [93]. Comprehensive lipidome analysis of the human tear fluid identified 17 major lipid classes with more than 600 lipid species. Besides the abundant phospholipids and sphingolipids, Lam et al., have identified and quantified cholesteryl sulfate, O-acyl-omega hydroxyl fatty acids as the amphiphilic lipid sublayer constituents [94]. Tear lipidome analysis in patients with dry eye syndrome (DES) showed positive correlation with levels of tear cholesteryl sulfate and glycophospholipids with physiological secretion of tears. Besides, reduced levels of low molecular weight wax esters and saturated fatty acyl moieties were observed in tears of DES patients and showed significant correlation with clinical parameters of DES as ocular surface disease index (OSDI), tear breakup time (TBUT) and Schirmer’s test I [95].

While tears immediately reflect the changes occurring at the cornea eyelids, aqueous humor or lachrymal glands alterations in tear constituents also reflects the disease mediated changes happening in the posterior portion of the eye, e.g. glaucoma. Differential lipidome analysis of aqueous humor in patients with open angle glaucoma (OAG) and controls (n = 10 each), revealed higher concentrations of diacylglycerophosphocholines and 1-ether 2-acylglycerophosphocholines in OAG patients compared to controls. Higher levels of sphingomyelins (SM) and cholesteryl esters are also observed in OAG compared to controls indicating increased lipid metabolism [96]. Increased lipid metabolism has been reported to be a consequence of higher oxidative ER-stress [97, 98] which might be playing a crucial role in the pathophysiology of open angle glaucoma. Sphingomyelins (SM) and phosphocholines (ceramides) have earlier been implicated with the pathophysiology of OAG [99].

#### Amniotic fluid lipidomics

The amniotic fluid lipidome has been studied in human pregnancy [100], to understand the lipid changes in spontaneous preterm [101, 102] and term birth [103], in clinical chorioamnionitis at term [104], fetal hyperlipidemia [105] and respiratory distress syndrome [106, 107] to name a few.

Using techniques of thin layer chromatography and gas chromatography, Singh and Zuspan have isolated and characterized the lipid content of human amniotic fluid from 24 weeks of gestation till labor. An increase in total lipid content (11.7 ± 1.0 mg to 15.1 ± 1.8 mg) was observed during this period. Phospholipids, free fatty acids, cholesteryl esters and hydrocarbons showed a predominant increase in their amounts by labor. The significant increase in phospholipids has been hypothesized to be due to the contribution of tracheal fluid during the period from gestation to labour. Also since phospholipids have a surfactant role, their increased levels is believed to assist during child birth or parturition. The ratio of phospholipids, especially lecithin/sphingomyelin (L/S ratio) is believed to be an indicator in the diagnosis of fetal maturity [100]. However L/S ratio was considered unreliable especially in cases with complicated pregnancy. Torday et al., introduced saturated phosphatodylcholine concentration as a predictor of fetal respiratory distress syndrome [106] with more sensitivity and specificity (1% false negatives). Shimizu et al., have developed a rapid (30 min) and sensitive method (2–65 mg/L) for quantitative determination of L/S ratio by fast bombardment mass spectrometry [107] from amniotic fluid samples (n = 10) which correlated with earlier methods of thin layer chromatography. This can help in better and accurate diagnosis of fetal maturity from amniotic fluid. Excess concentration of lipids was also observed in fetal hyperlipidemia. Elevated levels of cholesterol and triglycerides were observed in the amniotic fluid at 42 week gestation and subsequently in the 2-month-old infant indicating the possible role of cholesterol and triglycerides as indicators for prenatal diagnosis of hyperlipidemia [105].

Metabolic changes in preterm and term birth can also be studied from the lipidome of amniotic fluid. Gas chromatography mass spectrometry (GCMS) and liquid chromatography mass spectrometric (LCMS) analysis of the amniotic fluid from patients of spontaneous preterm birth and normal birth (n = 25 each) identified around 350 metabolites of which 116 metabolites belonging to hepatic metabolism, fatty acyl coA metabolism and histidine metabolism showed significant alteration in preterm births [101] indicating a gestation age effect on the metabolites. Amniotic fluid analysis of amniotic fluid of preterm women and paired maternal blood serum samples (n = 35 each) showed increased levels of lipids and altered metabolites in maternal serum of preterm births [102]. Alternately, quantitative LCMS analysis of amniotic fluid of patients at term (mid trimester), not in labor and those at term in spontaneous labor revealed significant increase in lipid mediators involved in epoxygenase-, lipoxygenase pathways in at term spontaneous labor [103]. However the physiological role of these lipid mediators during child birth needs to be further studied.

However, comparative lipidome analysis of spontaneous labor patients with clinical chorioamnionitis at term and without chorioamnionitis did not show significant difference in proinflammatory lipid mediators among the two groups whereas anti-inflammatory/proresolution lipid mediators were observed to be lower in patients with clinical chorioamnionitis indicating a significant inflammatory role in chorioamnionitis [104].

Table 1 depicts the list of lipids identified by mass spectrometry in major biological fluids and the disease association.

**Table 1:** List of lipids identified as probable diagnostic markers in biological fluids

S no	Biological fluid	Lipid class	Lipid species	Disease	Refs
1	Serum	Glycerophosphoserine (PS)	PS(O-16:0/20:2)	Hepatocellular carcinoma (HCC)	[62]
			PS(O-18:0/22:6)		
			PS(O-18:0/22:4)		
		Glycerophosphocholines (PC)	(PC(15:0/20:3))		
		Gycerophosophoinositols (PI)	PI(O-16:0/20:1)		
		Phospholipids/sphingomyelin (SM)	SM 16:0/1	Lung tumor	[64]
			LPC 18:1, 20:3, 20:4 and 22:6		
		Phospholipids	PC 38:5, 40:3 and 42:2	Prostate cancer	
			PC 36:9		[65, 66]
			FA 22:3		
2	Plasma		d18:1/14:0—SM	Gaucher’s disease	[63]
3	Urine	Triglycerides (TG)		Nephrotic syndrome	[80]
		Phosphatidylcholines (PC)			
		Phosphatidylethanolamine (PE)			
		Phosphatidylserine (PS)			
		Phosphatidylserine (PS)	PS 18:1/18:1 and 18:2/18:0	Breast cancer	[84]
		Phosphoinositol (PI)			
		Phosphatidylserine (PS)	PS 18:0/18:1	Prostate cancer	[85]
			PS 16:0/22:6		
			PS 18:1/18:0		
			PS 18:0/25:0		
4	Urine exosome	Phosphatidylserine (PS)	PS 18:1/18:1	Prostate cancer	[86]
		Lactosylceramide	d18:1/16:0		
			PS 18:0–18:2		
5	Cerebrospinal fluid	Glycerophospholipids (PL)		Alzheimer’s disease	[87]
		Sphingolipids (SL)			
6	Tear	Phospholipids (PL)		Dry eye syndrome	[95]
		Sphingolipids (SL)			
		Cholesteryl sulfate (CS)			
		Glycophospholipids			
7	Aqueous humor	Diacylglycerophosphocholines (DAG-PC)		Primary open angle glaucoma	[96, 99]
		1-ether 2-acylglycerophosphocholines (PC)			
		Sphingomyelins (SM)			
		Phosphocholines (PC)			

### Challenges in lipidome analysis

The diverse nature of the lipids poses certain challenges in carrying out lipidomics experiments. The major challenge that has to be overcome is extraction of lipids from the sample/specimen of choice. Due to their hydrophobic/amphipathic nature, diversity in structural and physicochemical properties among hundreds of thousands of lipids, a single extraction method does not yield the complete lipid repertoire. Tipthara and Thongboonkerd have demonstrated six different mixtures of solvent yield differential urinary lipid profiles unique to each combination of solvents [108]. A comprehensive, rapid method for extracting majority of the classes of lipids will help in overcoming the bias in lipid extraction and thereby can provide more comprehensive understanding of the lipid changes.

Another challenge in lipidomics is unambiguous identification of lipid species. Such challenges arise mostly incase of shotgun lipid analysis by direct infusion where orthogonal separation of lipids by liquid chromatography is not employed and are caused by isotopic species and adduct formations. Taking into consideration lipid databases as LIPID MAPS and high resolution/ultra-high resolution mass data, Bielow et al., have developed a new set of rules for true identification of lipids [109]. False identifications can also arise due to incorrect structural data in the lipid databases generated from means other than mass spectrometry. Liebsich et al., argue that structural data generated only by mass spectrometry should be used for reliable identification/annotation of lipid species. A detailed review by Liebsich et al, discusses on how a trustworthy lipidomics data has to be generated and reported [110].

These barriers when overcome along with advancements in mass spectrometry can carry lipidomics much deeper into the clinical scenario. Currently the mass spectrometry based lipidomics has been successful in identifying lipid markers for early detection of disease. The future application of such comprehensive lipidomic data from biofluids is likely to be point-of-care diagnostics for certain targets validated to be useful biomarkers in disease. Such lipid markers will then have utility for prognostication, rapid diagnostics and monitoring of treatment response. Furthermore, with advances in technological and data analysis platforms, the altered pathways can be targeted for treatment of the disease.

## Conclusion

Biofluids serve as a repertoire of information on the molecular changes occuring in health and in disease. A pan-omics systems biology approach helps in understanding the disease associated changes. Lipidomics provides a snapshot of the disease related changes in the metabolic pathways which might be directly related to the manifestation of the disease. Mass spectrometry based lipidomics analysis of biofluids provides the advantage of identifying accurate and relevant alterations in the molecules which can serve as diagnostic markers or drug targets.

## Abbreviations

APCI: atmospheric pressure chemical ionisation
CSF: cerebrospinal fluid
DES: dry eye syndrome
ESI: electrospray ionisation
GC: gas chromatography
LCMS: liquid chromatography mass spectrometry
NMR: nuclear magnetic resonance spectroscopy
OAG: open angle glaucoma
OSDI: ocular surface disease index
TBUT: tear breakup time
TLC: thin layer chromatography
